# THE EFFECTS OF LIFETIME EXPERIENCES OF HOMELESSNESS AND INCARCERATION ON COGNITIVE AGING

**DOI:** 10.1093/geroni/igac059.2473

**Published:** 2022-12-20

**Authors:** Douglas Hanes, Sean Clousten

**Affiliations:** Stony Brook University, Stony Brook, New York, United States; Stony Brook University, Stony Brook, New York, United States

## Abstract

Alzheimer’s disease and related dementias (ADRD) remain a pressing health concern in the U.S., and this burden and access to care are unequally distributed across the population. The U.S. has one of the highest incarceration rates globally, which is also unequally distributed; and groups like veterans, formerly incarcerated people, and sexual and gender minorities (SGM) face high risk of homelessness. Homelessness and incarceration are potentially traumatic experiences in themselves. Both experiences are more likely among people with less formal education, less economic security, and racialized groups, even as they reduce educational and economic opportunities. Using data from the Health and Retirement Study (HRS; 1998–2016), we investigate whether self-reported lifetime experiences of homelessness and incarceration (including time spent incarcerated) are associated with cognitive functioning and risk of possible dementia. Multilevel modeling adjusted for age, education, and other demographic covariates revealed that lifetime experiences of homelessness and incarceration are associated with lower cognition (Homelessness: β=-1.231, p< .001; Incarceration: β=-0.929; p < .001), but slower aging-related declines (Homelessness-slopes: β=0.044, p<.001; Incarceration-slope β=0.041; p<.001), and homelessness moderated the impact of prior incarceration (β=-1.789; p<.001), but less-steep declines (β=0.092; p <.001). Homelessness and incarceration, independent of their other risk factors and associated harms, have associations with ADRD risk.

